# Lignin Extraction from Date-Palm Waste for Structural and Thermal Applications: A Comparative Study of Alkaline and Deep Eutectic Solvent Pretreatments

**DOI:** 10.1021/acsomega.5c09123

**Published:** 2025-11-17

**Authors:** Alaa A. Dandash, Joy H. Tannous, Basim Abu-Jdayil

**Affiliations:** † Department of Chemical and Petroleum Engineering, 11239 United Arab Emirates University, Al-Ain, United Arab Emirates; ‡ National Water and Energy Center, United Arab Emirates University, PO BOX 15551, Al Ain 15551, United Arab Emirates

## Abstract

Lignin, a major component of abundant wood from date-palm waste, remains largely underutilized despite its potential as a valuable bioresource. The improper disposal of this waste contributes to environmental hazards, underscoring the need for sustainable valorization strategies. While prior studies have explored lignin extraction from different biomasses using either deep eutectic solvents or alkaline methods, limited work has provided a direct comparison of these techniques on the same date-palm waste feedstock, nor has the effect of the deep eutectic solvent pretreatment time on lignin characteristics been systematically investigated. This study addresses these gaps by evaluating lignin extraction from date-palm waste using a green deep eutectic solvent composed of choline chloride and formic acid, while varying pretreatment time (2–8 h) and alkaline pretreatment methods. The extractions’ performances were evaluated by calculating lignin yield and purity. The extracted lignins were characterized using FTIR, TGA, DSC, SEM, XRD, molecular weight determination, and ESR. While the deep eutectic solvent lignin exhibited better purity—up to 63.44%, alkaline pretreatment produced a higher lignin yield of 36.7% but with a considerably reduced purity of 27.42%. SEM and FTIR results verified that the deep eutectic solvent lignin maintained a more intact molecular structure with less carbohydrate fragmentation. Moreover, TGA results indicated that the deep eutectic solvent lignin exhibited enhanced thermal stability. A deep eutectic solvent pretreatment time of 4 h was found to be optimal, yielding the highest lignin yield (22.85%) with high purity (58.89%). Extending the duration to 8 h led to increased fragmentation, a reduced molecular weight (4.90 kDa), and a slight decline in thermal stability due to possible degradation. These results highlight the potential of deep eutectic solvent pretreatment as a sustainable substitute for conventional lignin extraction methods to produce high-purity, thermally stable lignin.

## Graphical abstract

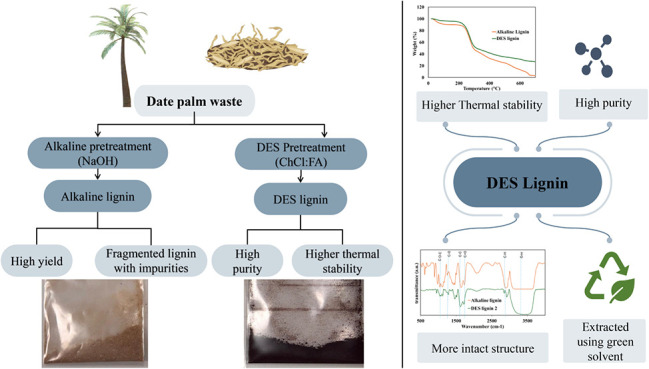

## 1 Introduction

The increasing demand for sustainable materials, coupled with the depletion of fossil resources and the increasing effects of climate change, has intensified global efforts to reduce reliance on fossil resources. This shift has led to worldwide initiatives to transition to renewable and eco-friendly alternatives.^1^ In this context, the global energy sector is promoting the valorization of lignocellulosic biomass into high-value and sustainable bioproducts, as it represents a promising pathway toward a circular bioeconomy.^2–5^ According to the International Energy Agency, lignocellulosic biomass could supply 10% of the world’s energy by 2035.^6^ In addition to its energy potential, converting biomass into biofuels and materials helps lower carbon emissions and enables the production of commercially valuable, high-added-value products from renewable resources.^7^

Lignocellulosic biomass comprises three main components: cellulose, hemicellulose, and lignin, each of which can serve as a valuable platform for biobased products. The composition and relative proportions of these components are highly dependent on the biomass source.^8^ Over the past few decades, researchers have been attempting to fractionate these components so that they can be used to produce various biofuels and chemicals.^9,10^

Among different biomass sources, date-palm waste (DPW) is an abundant but underutilized lignocellulosic resource. Mostly generated from agricultural pruning and date-fruit-gathering activities,^11^ the United Arab Emirates (UAE) produces ∼500,000 tons of DPW annually. Unfortunately, much of this waste ends up being burned, buried, or left to decompose, leading to environmental problems such as ineffective resource utilization and greenhouse gas emissions.

Despite being the second-most abundant biopolymer on Earth, lignin remains an underutilized resource, produced primarily as waste in the paper- and pulp-processing sector or as a byproduct in bioethanol production.^12^ Recently, lignin has attracted remarkable attention, especially owing to its potential in developing sustainable alternatives to petroleum-derived products and possible applications in biobased compounds and chemicals, energy storage, and polymer synthesis.^13,14,15^ The distinctive characteristics of lignin make it a highly versatile polymer, enabling its use across a wide range of applications. These applications include its use in thermal-insulation aerogel synthesis^16^ as an antioxidant agent^17^ and in wood adhesives.^18^ In addition, lignin can be employed in pharmaceuticals, such as sunscreens, as its addition enhances UV protection.^19^ Furthermore, lignin valorization has recently expanded toward food packaging and agricultural applications, where its biodegradability, antioxidant, and antimicrobial properties contribute to the development of sustainable and active materials that can enhance food preservation and soil health.^20,21^

One of the major challenges in lignin extraction lies in its complex, heterogeneous structure and strong interactions with cellulose and hemicellulose, which require efficient pretreatment strategies to isolate lignin in high yield and purity without severe degradation. Conventional lignin extraction techniques often utilize alkaline pretreatment, employing sodium hydroxide (NaOH) or other alkaline chemicals to disrupt the lignin–carbohydrate bonds and thus solubilize lignin. However, alkaline extraction has notable disadvantages, such as the production of wastewater containing residual alkali and insufficient effectiveness in pretreating biomass feedstocks with high lignin content. In addition, alkaline pretreatment may degrade the lignin structure, which may restrict its utilization in diverse applications.^8,22^

Although lignin extraction from biomass, such as bamboo,^18^ sugarcane bagasse,^23^ and rice waste,^24^ has been studied extensively, its extraction from DPW remains relatively underexplored. To date, investigations have primarily focused on the Klason method, including acidic and alkaline pretreatments, for lignin extraction from DPW.^25,26^ A study of five types of DPW generated from palm trees in the Gabes oasis, Tunisia, where lignin was extracted using acidic pretreatment, reported lignin yields ranging from 22.54% to 31.63%, with palm fronds exhibiting the highest yield. The recovered lignin was further utilized as an additive in preparing low-density polyethylene membranes for treating oily wastewater.^27^ Although pretreatment via acid hydrolysis enables rapid lignin separation with high hydrolysis rates, this method presents considerable challenges due to the highly toxic and corrosive nature of acidic reaction media, requiring the implementation of resistant and durable reactors; this implementation, in turn, increases the biomass-processing costs.^28^ In contrast, green solvent systems, such as deep eutectic solvents, offer significant environmental and sustainability advantages, including the elimination of hazardous acids, reduced energy consumption, and minimized secondary waste generation, making them a cleaner and safer alternative for large-scale biomass valorization. The gap in research on green solvents for lignin extraction from DPW motivated us to examine a green pretreatment method for lignin extraction from DPW as an alternative to conventional acidic pretreatment.

Deep-eutectic solvent (DES) pretreatment represents an effective, sustainable, and ecologically friendly alternative for lignin extraction. DES synthesis utilizes low-cost, readily accessible materials. In addition, DES pretreatment has advantages over other lignin extraction techniques, including low volatility, a broad liquid range, relatively high biodegradability, and compatibility with enzymes.^29^ Compared to conventional lignin extraction methods, DES pretreatment exhibits enhanced selectivity for lignin dissolution throughout the pretreatment process owing to the ability of DES to break aryl–ether bonds (β-O-4) and specific lignin–carbohydrate covalent bonds.^30,31^ The use of DES pretreatment to extract lignin results in increased yields, favorable purity, and reduced molecular weight, which are advantageous for future applications.^32,33^ Pretreatment time plays a critical role in DES-based lignin extraction, as prolonged reaction durations enhance lignin solubilization and cleavage of ether linkages, which increases extraction yield; however, excessively long pretreatment may promote condensation or degradation reactions, reducing lignin quality and recovery efficiency. Therefore, an optimized pretreatment time is essential to balancing lignin yield, structural integrity, and process sustainability. A mixture of choline chloride and lactic acid (ChCl:LA) is identified as an effective DES capable of selectively dissolving 90% of lignin from raw eucalyptus.^31^ A study indicated that under optimal conditions, up to 81.1% (at 160 °C for 13.12 h) and 82% (at 160 °C for 14.04 h) of lignin present in sugarcane bagasse were extracted using choline chloride: glycerol (ChCl:Gly) and choline chloride:urea (ChCl:urea), respectively.^34^ Another study compared lignin extraction from *Eucalyptus tenuifolia* sawdust via pretreatment with two DESs—one consisting of ChCl mixed with formic acid (FA) and the other comprising ChCl mixed with lactic acid (LA). The results showed that the FA-based DES system demonstrated superior efficiency in lignin extraction.^35^ Based on these findings, this study employs a ChCl:FA DES for lignin extraction from DPW and analyzes the characteristics of extracted lignin. While numerous studies have investigated lignin extraction from lignocellulosic biomass utilizing DESs,^36^ this study addresses the research gap regarding the impact of pretreatment time on the thermal and structural characteristics of the extracted lignin.

This study aims to enhance biomass valorization by developing and optimizing an eco-friendly lignin extraction process. The novelty of this work lies in exploring, for the first time, the use of DES for lignin extraction from abundantly available DPW in the UAE, presenting a sustainable alternative to conventional extraction techniques. Furthermore, this research provides a direct comparative assessment between DES and alkaline pretreatments applied to the same biomass, supported by comprehensive material characterization to evaluate their potential in advanced biobased applications such as thermal insulators and polymers. The study also seeks to optimize the DES pretreatment duration to achieve maximum lignin yield and purity.

## 2 Experimental Section

### 2.1 Materials

DPW was sourced from the Al-Foah Farm of the UAE University, located in Al Ain, Abu Dhabi, United Arab Emirates (coordinates: 24.35896, 55.79922). The raw material was ground using a commercial milling machine and then mechanically sieved to obtain a powdered biomass with a grain size of 150 μm. The chemical composition of the DPW was determined following the procedure described in a previous experimental study,^37^ which included the determination of extractives, holocellulose, α-cellulose, and lignin, employing standardized analytical methods established by the Technical Association of the Pulp and Paper Industry (TAPPI). Extractives were quantified using TAPPI T 204 cm-97, α-cellulose following TAPPI T 203 cm-99, and lignin using TAPPI T 222 cm-99. The holocellulose content was determined according to the method of Wise et al.,^38^ in which lignin is selectively removed from wood through acidified sodium chlorite delignification to yield a holocellulose fraction composed of cellulose and hemicellulose. Although the original study did not involve enzymatic hydrolysis, subsequent research utilizing the Wise method commonly applies cellulase at 5 FPU g^–1^ substrate and β-glucosidase at approximately one-ninth of the cellulase activity when evaluating the enzymatic digestibility of delignified biomass. Hemicellulose content was calculated by subtracting α-cellulose from the holocellulose fraction. The chemical composition of the DPW used in this study was found tobe 10.4% extractives, 39.8% cellulose, 30.6% hemicellulose, and 17.1% lignin.

Commercial lignin (kraft lignin) was purchased from Sigma-Aldrich and characterized to compare its functional and thermal characteristics with those of the extracted lignins. In addition, other chemicals utilized in this study—sodium hydroxide (NaOH, ≥97.0%, pellets), sulfuric acid (H_2_SO_4_, 95–97%), ChCl (≥98%), FA (≥95%), potassium bromide (KBr, FTIR reagent, ≥99.0%), and 1,4-dioxane (ACS reagent, ≥99%)—were procured from Sigma-Aldrich. Ethanol (absolute, analytical grade) was sourced from Merck (Germany). Technical calibration buffer solution (pH 6) was sourced from Hanna Instruments. Additionally, filtration was performed using Whatman grade GF/C glass microfiber filter disks with a 1.2-μm pore size.

### 2.2 Equipment and Procedures

#### 2.2.1 Extraction of Lignin Using Alkaline Pretreatment

A 2 M (8%) sodium hydroxide (NaOH) aqueous solution was prepared by dissolving NaOH in deionized water. A total of 500 mL of the prepared NaOH solution was mixed with 10 g of previously ground and milled DPW powder. This mixture was then stirred continuously at 80 °C for 2 h, in alignment with mild extraction conditions reported in literature,^39,40^ to avoid extensive degradation of the lignin structure. Following pretreatment, the slurry was vacuum-filtered to separate the black liquor containing solubilized lignin from the solid residue. The extraction procedure was repeated on the same solid biomass until the filtrate was colorless, indicating the total removal of lignin. The black liquor was preserved for lignin precipitation in the next few steps.

A 2.5 M H_2_SO_4_ solution was prepared and gradually introduced into the black liquor until its pH was reduced to 2 to induce the precipitation of acidified lignin. The mixture was left in the fridge overnight to allow precipitation. The resulting suspension was centrifuged using a Hermle benchmark centrifuge (Z366 K) at 3600 rpm for 15 min to separate lignin particles, which were then collected and rinsed with distilled water. The washed lignin suspension was centrifuged again under the same conditions (i.e., 3600 rpm for 15 min) to further precipitate lignin from the solution. The recovered lignin was then freeze-dried using a Manifold Freeze-Dryer LBFD-D11 for 24 h to obtain it in powdered form.

#### 2.2.2 Extraction of Lignin Using DES Pretreatment

A DES was prepared using ChCl, which was dried at 80 °C for 6 h and then allowed to cool to an ambient temperature of 25 °C in a desiccator. FA and ChCl were mixed in a 1:2 molar ratio inside sealed glass bottles and heated to 70 °C under continuous stirring until a clear, transparent liquid was produced. To enable biomass fractionation,10 g of DPW was then combined with 150 mL of the prepared DES at a 1:15 mass ratio in a sealed glass bottle and stirred at 120 °C for varying pretreatment times of 2, 4, 6, and 8 h. The resulting mixture was then filtered to separate the filtrate from the solid residue. The solid was repeatedly washed with ethanol until the wash solution flowed clear. To recover lignin from the DES solution, an ethanol: water (1:9) mixture, whose volume was three times the filtrate volume, was added to the filtrate as an antisolvent, and this suspension was then thoroughly stirred for 1 h and left overnight to precipitate lignin from the filtrate. The precipitated lignin was centrifuged using the Hermle benchmark centrifuge (Z366 K) at 3,600 rpm for 15 min to further precipitate lignin particles and separate them from the antisolvent mixture. The precipitated lignin was then repeatedly washed with aqueous ethanol and centrifuged. The pure lignin was then freeze-dried using a Manifold freeze-dryer LBFD-D11 to produce a fine powder. Figure 1 summarizes the flow diagram of the lignin extraction process using DES.

**Figure 1:**
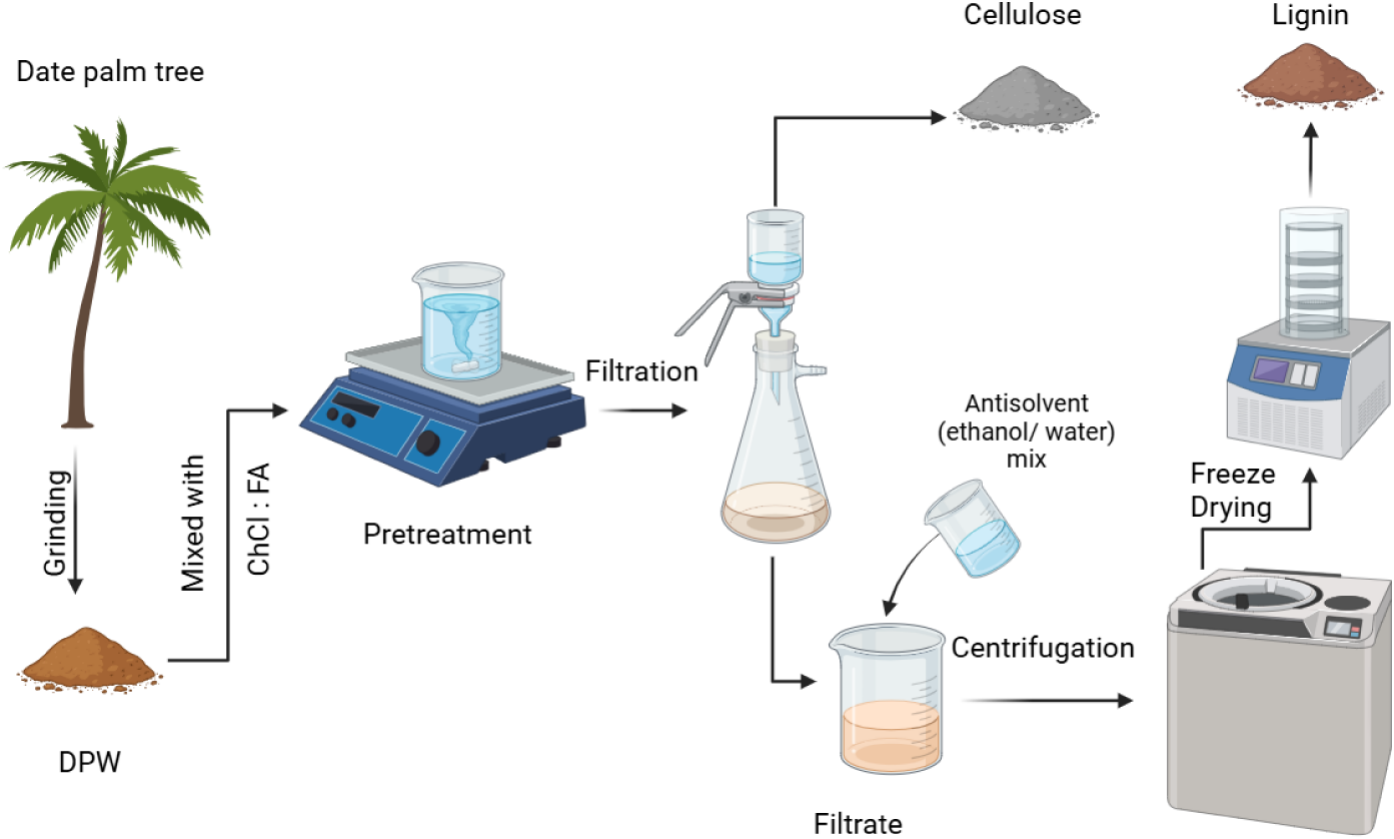
Illustration of the deep eutectic solvent extraction process for lignin.

#### 2.2.3 Lignin Purity

The purity of the extracted lignin was analyzed using the NREL method for determining structural carbohydrates and lignin present in biomass.^41^ For this purpose, 0.3 g of dehydrated biomass was hydrolyzed using 3 mL 72% H_2_SO_4_ at 30 °C for 1 h under occasional stirring to ensure the even exposure of biomass to H_2_SO_4_. Subsequently, the acid was diluted by adding 84 mL of distilled water, lowering the H_2_SO_4_ concentration to 4%. The mixture was then autoclaved at 121 °C for 1 h to ensure complete hydrolysis. Further, the mixture was filtered to separate the solid residue, which represents the acid-insoluble lignin (AIL), and to collect the filtrate, which denotes the acid-soluble lignin (ASL). ASL was analyzed utilizing a SPECORD 200 PLUS double-beam spectrophotometer, with its UV–vis spectrum obtained using 1% H_2_SO_4_ as a blank to calibrate the spectrophotometer and by measuring absorbance at a wavelength of 205 nm.

#### 2.2.4 Lignin Characterization

The lignins extracted using both pretreatment methods were characterized to elucidate and compare their physical, chemical, and morphological properties.

##### 2.2.4.1 Fourier Transform Infrared (FTIR) Spectroscopy

Functional groups in the lignin samples were detected using FTIR spectroscopy, based on which the influence of the two biomass pretreatments on the lignin chemical composition was investigated. A Thermo/Nicolet Nexus 470 ESP FTIR spectrometer (USA) was used to collect the spectra in the wavenumber range of 500–4000 cm^–1^. The samples were ground, combined with KBr, and compacted into disks before analysis to enable precise spectrum collection.

##### 2.2.4.2 Scanning Electron Microscopy (SEM)

A JEOL JCM-5000 NeoScope scanning electron microscope was employed to investigate the morphological properties of the raw untreated DPW, commercial lignin, and extracted lignin samples. To enhance the imaging quality and minimize electrostatic charging during analyses, the samples were coated with a thin layer of gold through sputtering. The high-resolution images of these samples were obtained via SEM at an acceleration voltage of 5 kV.

##### 2.2.4.3 Thermogravimetric Analysis (TGA)

The thermal stability and decomposition properties of the raw untreated DPW, commercial lignin, and extracted lignin samples were assessed via TGA. At a nitrogen flow rate of 100 mL/min, the analysis was performed using a PerkinElmer TGA 7 instrument (USA) under a controlled nitrogen atmosphere. The samples (amount: 5–10 mg) were subjected to nonisothermal heating from 30 to 900 °C at a heating rate of 5 °C/min.

##### 2.2.4.4 Differential Scanning Calorimetry (DSC)

Using a differential scanning calorimeter (Discovery 25, TA Instruments, USA), we investigated the thermal transition behaviors of the commercial, alkaline, and DES lignin samples. To ensure proper thermal characterization, the sample amount for DSC analysis varied from 5 to 10 mg within a controlled temperature range of 25 °C–270 °C. The analysis was performed at a consistent heating rate of 10 °C/min under a stable nitrogen atmosphere, with a consistent flow rate of 50 mL/min.

##### 2.2.4.5 X-ray Diffraction (XRD) Analysis

The XRD analyses of raw untreated DPW, commercial lignin, alkaline lignin, and DES-extracted lignins after different pretreatment times were conducted to examine the overall crystalline phases in these samples. The analysis was performed by utilizing a Malvern Panalytical X-ray diffractometer. The anode material was copper, and the θ value varied from 10° to 40°.

##### 2.2.4.6 Determination of the Average Molecular Weight of Lignin

The average molecular weights of the lignin samples were estimated based on intrinsic viscosity, as reported in previous studies.^42,43,44^ All lignin samples were dissolved in dimethylformamide (DMF), and their viscosity values were measured using an Anton Paar SVM 3001 viscometer at different concentrations.

##### 2.2.4.7 Phenolic Hydroxyl Content Determination

The total phenolic hydroxyl (PhOH) content of the alkaline-extracted lignin, the optimum DES lignin extracted at 4 h, and commercial lignin was determined by UV spectrophotometry as described by Tan et al.^45^ One milligram of lignin sample was dissolved in 5 mL of 1,4-dioxane and 5 mL of 0.2 M NaOH, and then the solution was filtered to remove particulates. After that, two solutions were prepared for the absorbance measurement. Solution A (Reference): 2 mL of lignin solution was diluted to 25 mL with pH 6 buffer, and Solution B (Sample): 2 mL of lignin solution was diluted to 25 mL with 0.2 M NaOH. Solution B’s UV–vis absorbance spectrum was recorded at 300 and 360 nm, using Solution A as the reference.

##### 2.2.4.8 Free-Radical Investigation Using Electron Spin Resonance (ESR)

ESR spectroscopy was performed using Bruker ESR-5000 equipment operating at 100 kHz. The magnetic field was swept from 332 to 340 mT over 60 s. The DPW, commercial lignin, alkaline lignin, and DES lignin samples were analyzed at 298 K. Each sample was prepared by placing it in a 5 mm diameter synthetic quartz ESR tube (Bruker ESR-5000 P165/5) with a sample height of 32 mm to ensure proper detection by the resonant cavity of the ESR spectrometer. The experimental spectra were compared by performing double integration to quantify the signal intensities. The ESR spectrometer configuration was optimized by adjusting the microwave power, modulation amplitude, and power saturation to account for sample differences.

### 2.3 Calculations

#### 2.3.1 Yield and Purity of the Extracted Lignin

The yield and purity of the extracted lignin were calculated using equations reported previously.^46^ The lignin yield was determined to evaluate the efficiency of the extraction process. The yield was calculated using eq 1 as follows:

1 $$\mathrm{Yield}=\frac{\mathrm{amount}\;\mathrm{of}\;\mathrm{extracted}\;\mathrm{lignin}}{\mathrm{amount}\;\mathrm{of}\;\mathrm{lignin}\;\mathrm{in}\;\mathrm{biomass}}\times 100$$

where the “amount of extracted lignin” refers to the dry weight of lignin obtained after the extraction process, and “amount of lignin in biomass” represents the lignin content in raw, untreated DPW before extraction.

For lignin purity calculation, after filtering the sample and drying the solid residue, the weighed AIL was calculated using eq 2 The ASL concentration in the filtrate was determined based on its absorbance value at a wavelength of 205 nm, obtained using a UV–Vis spectrophotometer, to estimate ASL% using eq 3. The sum of the AIL and ASL concentrations gave the total lignin content in biomass, as shown in eq 4:

2 $$\%\mathrm{ASL}=\frac{{\mathrm{UV}}_{\mathrm{abs}}\times {\mathrm{Volume}}_{\mathrm{filterate}}\times \mathrm{Dilution}}{\varepsilon \times {\mathrm{ODW}}_{\mathrm{sample}}\times \mathrm{Path}\;\mathrm{length}}\times 100$$

3 $$\%\mathrm{AIL}=\frac{\mathrm{Dry}\;\mathrm{residual}\;\mathrm{weight}}{\mathrm{Initial}\;\mathrm{biomass}\;\mathrm{weight}}\times 100$$

4 LigninPurity=%AIL+%ASL

Where UV_abs_ is the absorbance of the filtrate measured by UV-vis, ε is the molar absorptivity coefficient of lignin (L/g·cm), and ODW_sample_ is the oven-dry weight of the untreated raw DPW (g).

All experiments were conducted in triplicate, and the data for extraction yields and purity are presented as the mean ± standard deviation (SD). One-way analysis of variance (ANOVA) was performed to evaluate statistical significance among different treatment groups. A p-value of less than 0.05 was considered statistically significant.

#### 2.3.2 Crystallinity Index of the Cellulosic Impurities in Extracted Lignin

The crystallinity index (CrI%) was estimated as previously calculated in^37^ using the Segal eq 5:

5 $$\mathrm{CrI}\%=\left(I_{\mathrm{cry}}-I_{\mathrm{amp}}\right)/I_{\mathrm{cry}}\times 100$$

Where I_cry_ represents the highest peak intensity (crystalline region) and I_amp_ signifies the diffraction intensity for the amorphous region.

#### 2.3.3 Determination of the Average Molecular Weight of Lignin

Intrinsic viscosity (*η*) (mL/g) for each sample was determined by fitting the concentration and viscosity data for different lignin concentrations using the Huggins equation (eq 6). The specific viscosity divided by concentration *(η*_sp_/c) was plotted against c. [*η*] was then deduced from the y-intercept as follows:

6 $$\eta_{\mathrm{sp}}=\left[\eta \right]+k_{H}{[\eta ]}^{2}c$$

Where *η*_sp_ is the specific viscosity of the lignin solution (unitless), c is the lignin concentration in the solution (g/mL), and k_H_ is the Huggin’s coefficient (empirical constant) (unitless)

The molecular weight of lignin was then determined using the Mark–Houwink formula (eq 7) based on [*η*]:

7 $$\left[\eta \right]={{\mathrm{KM}}_{w}}^{a}$$

where M_w_ is the molecular weight of lignin (kDa), and K = 0.11 and a = 2.51 (constants specific to lignin in DMF).

#### 2.3.4 Phenolic Hydroxyl Content Determination

The phenolic hydroxyl content (PhOH) was calculated by using eq 8:

8 $$\mathrm{PhOH}(\mathrm{mmol}/g)=\left(0.250\times \Delta a_{300,\mathrm{NaOH}}\right)+\left(0.107\times \Delta a_{360,\mathrm{NaOH}}\right)$$

Where Δ*a* = absorbance × path length/(lignin solution concentration (mg/mL)).

## 3 Results and Discussion

### 3.1 Analysis of the Yield and Purity of the Extracted Lignin

This study comprehensively examined the effects of alkaline and DES pretreatments on the yield and purity of the lignin extracted from DPW. In addition, the impacts of these pretreatment methods on the lignin’s structural and thermal characteristics were analyzed. One alkaline pretreatment was performed for comparison, and four DES pretreatments were conducted by using the same DES but with different pretreatment times to further explore the impact of pretreatment time on DES-based lignin extraction. Each experiment was performed three times to ensure the repeatability of our results. Section 3.1 provides an in-depth discussion of the findings, examining variations in lignin yield and purity across different pretreatment methods and conditions and offering insights into the effectiveness of each extraction method.

The bar chart presented in Figure 2 shows the yield and purity of lignin extracted using alkaline pretreatment and DES pretreatment for different times (2, 4, 6, and 8 h). Statistical analysis using one-way ANOVA revealed significant differences in the lignin yield across the different pretreatments (*p* = 1.66 × 10^–5^). The lignin yield obtained using alkaline pretreatment is the highest compared to all lignin samples extracted via DES pretreatment. The alkaline lignin exhibits the highest yield of 36.7%, while the highest yield obtained via DES pretreatment reaches 22.88%. Similarly, lignin purity also varied significantly across treatments (*p* = 1.78 × 10^–8^). The alkaline lignin has a lower purity than all DES samples, at 27.42%. The DES lignins extracted after different pretreatment times demonstrate a specific trend: purity decreases slightly as the pretreatment time increases, with the DES lignin pretreated for 2 h exhibiting the highest purity of 63.44%. High purity is consistently observed across all pretreatment times. These statistically significant results demonstrate the distinctive effects of DES and alkaline pretreatments in lignin extraction.

**Figure 2:**
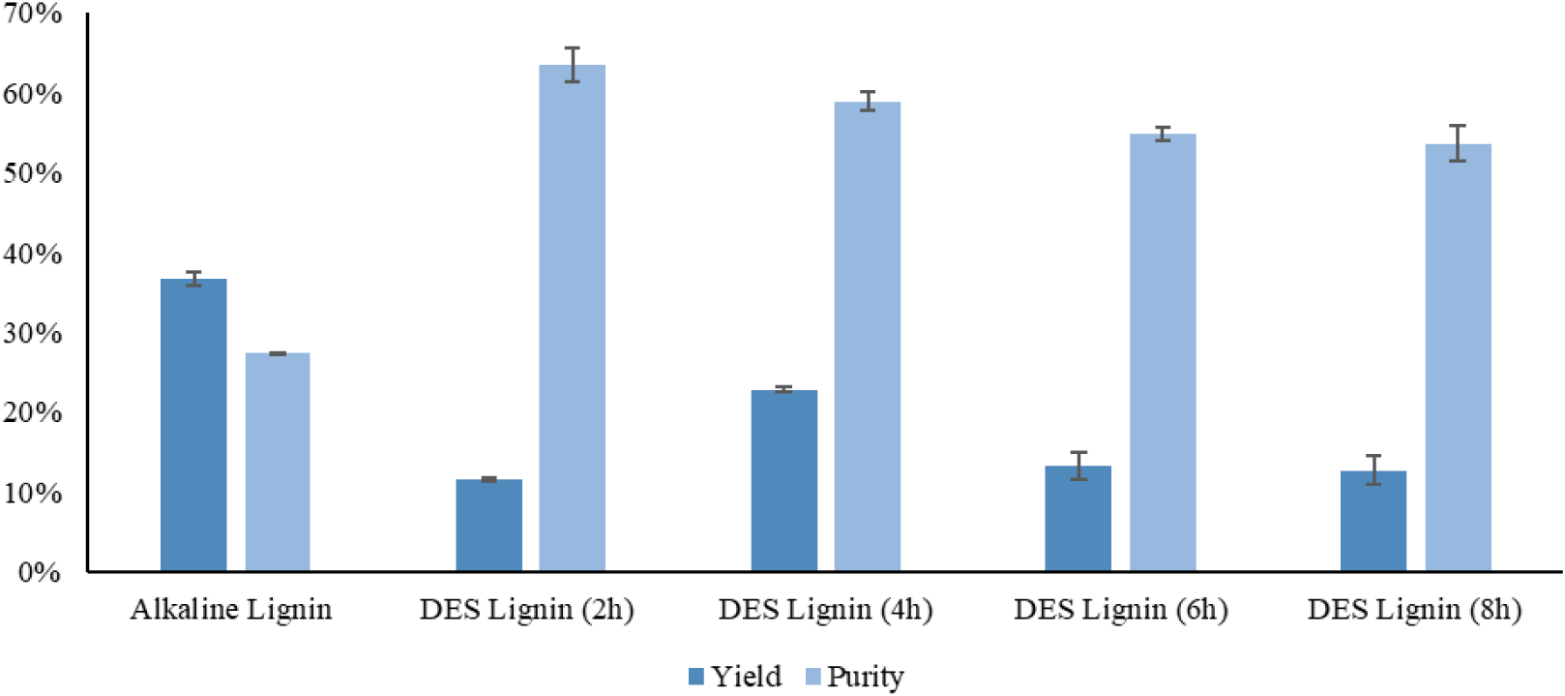
Yield and purity of alkaline lignin and DES lignin at different pretreatment times of 2, 4, 6, and 8 h. Error bars show the standard deviation of triplicate samples.

The high yield and low purity of the alkaline lignin indicate that, while alkaline extraction is efficient in extracting lignin, this method also extracts impurities such as hemicellulose and cellulose, thereby reducing overall purity. This aspect will be further elaborated upon when discussing the different characterization results of all extracted lignins, with results highlighting whether the alkaline lignin yield is indeed lignin or whether it contains other constituents. Meanwhile, the DES lignins display higher purity, ascribed to the chemical composition of the DES.

Acidic DESs derived from natural components, such as formic acid, have been reported to demonstrate significant efficiency in fractionating lignocellulosic biomass and high selectivity for lignin while enhancing cellulose accessibility.^47^ The synthesized acidic DES in this study comprises a hydrogen-bond acceptor, specifically FA, and a hydrogen-bond donor (HBD), namely ChCl.^33^ The selective solubilization of lignin using ChCl:FA-based DESs can be primarily attributed to their acidity and hydrogen bond-donating ability. During DES pretreatment, halide anions from the HBA, such as the chloride ion in choline chloride, form hydrogen bonds with hydroxyl groups on lignin, facilitating lignin dissolution and the extraction of phenolic groups.^48^ The acidic hydrogen-bond donor (HBD), such as formic acid, enhances the cleavage of β-O-4 ether linkages and lignin–carbohydrate bonds, enabling the recovery of low-molecular-weight lignin with high purity.^49^ In spite of the high selectivity for lignin, the acidic conditions employed often lead to considerable hemicellulose degradation.^47^ Therefore, the ChCl:FA DES used in this study demonstrates relative selectivity for lignin over carbohydrates.

The purity of the lignin extracted using different DESs ranged from 70% to 85.53%, which is higher than that reported for alkaline and acidic lignin (i.e., 49.4% and 31.36%, respectively).^19,27,42,51,52^ However, pretreatment time optimization performed in this study resulted in a higher lignin yield of 22.66% after pretreatment for 4 h, surpassing the previously reported yield of 16.94% after 3 h, wherein the same DES was used to extract lignin from bamboo.^50^

Figure 2 depicts that a higher lignin yield is recovered using the DES pretreatment as the time increases, with the maximum lignin yield being acquired at 4 h. This may be due to the fact that as the pretreatment time increases, the DES–biomass contact enhances, leading to better fractionation of the lignocellulosic biomass structure and, consequently, a higher lignin yield.^34^ However, when the pretreatment time is extended, the yield decreases, possibly due to excessive degradation or condensation reactions that limit lignin extraction. The SEM images for DES lignin at 6 and 8 h further support these findings, revealing a more fragmented and disrupted structure indicative of excessive degradation at prolonged pretreatment times.

These results highlight the efficiency of the DES pretreatment method in extracting high-purity lignin, especially exhibiting a purity of 58.89% and an optimal extracted lignin yield of 22.85% under 4 h of pretreatment time.

In addition, a statistical analysis was performed for yield and purity. It was found that the p-value for yield is 1.659 × 10^–5^ that is less than 0.05, indicating a highly significant difference between extraction methods. The p-value for purity was found to be 1.78 × 10^–8^ implying an extremely significant difference in terms of the purity of the extracted lignin. Therefore, the differences in yield and purity of the extracted lignin among the different methods are statistically significant—the observed variations are very unlikely to be due to random chance.

### 3.2 Structural Characterization of the Extracted Lignin Using FTIR Spectroscopy

The FTIR results are presented in this section, revealing the chemical structure and composition of the raw DPW biomass, commercial lignin, and lignin extracted by using alkaline and DES pretreatments. These results are used to compare structural modifications that occur when DES and alkaline pretreatments are employed for lignin extraction. FTIR spectroscopy provides insights into the functional groups present in lignin and highlights modifications resulting from different pretreatment conditions.

The FTIR spectra presented in Figure 3 exhibit similar patterns and are consistent with functional groups normally found in lignin, with some variations in band intensities. Figure 3A illustrates the infrared spectra for DPW, commercial lignin, alkaline lignin, and DES lignins. A dominant peak occurring in the range of 3200–3550 cm^–1^ is found in all samples; it corresponds to the stretching vibrations of the O–H bonds of hydroxyl groups in lignin.^53^ Increased band intensities exhibited by commercial and DES lignin suggest a higher number of aromatic structures in these samples. The bands at 2830 and 2965 cm^–1^ represent C–H stretching related to methyl aliphatic groups. The higher intensity of the C–H peak for the alkaline lignin indicates a higher degree of lignin depolymerization, as NaOH cleaves β-O-4 ether linkages and destroys the aromatic skeletal carbon of lignin, generating smaller lignin fragments.^54^ The C=O bond stretching peak observed at 1706–1720 cm^–1^ corresponds to the carbonyl stretching vibration, which is prominent in the DES lignin and alkaline lignin. This suggests the presence of carbonyl groups conjugated to aldehydes or ketones formed due to side-chain oxidation and β-O-4 linkage cleavage occurring during pretreatment.^55^ Highly intense peaks observed at 1600–1400 cm^–1^ Aromatic corresponding to aromatic C=C stretching, are exhibited by all samples, confirming the presence of the characteristic aromatic structure of lignin. The commercial lignin demonstrates sharper peaks, implying a more defined aromatic structure.^56^ The C–O stretching band observed at 1200–1275 cm^–1^ is primarily associated with ether linkages connecting lignin structural units as well as with the C–O stretching vibrations of phenolic hydroxyl groups. The C–O–C stretching peak appearing at 1037 cm^–1^ is ascribed to pentose, and the hexose-ring skeletal vibration peak at 897 cm^–1^ indicates the presence of β-glucosidic bonds in cellulose and hemicellulose. They are seen more in the raw DPW and alkaline lignin, indicating the presence of cellulose and hemicellulose in them.^57,58^ These observations suggest that the DES lignin possesses superior purity and is less contaminated by carbohydrate fragments compared to the alkaline pretreated lignin,^59^ consistent with the above purity analysis of the extracted lignin.

**Figure 3:**
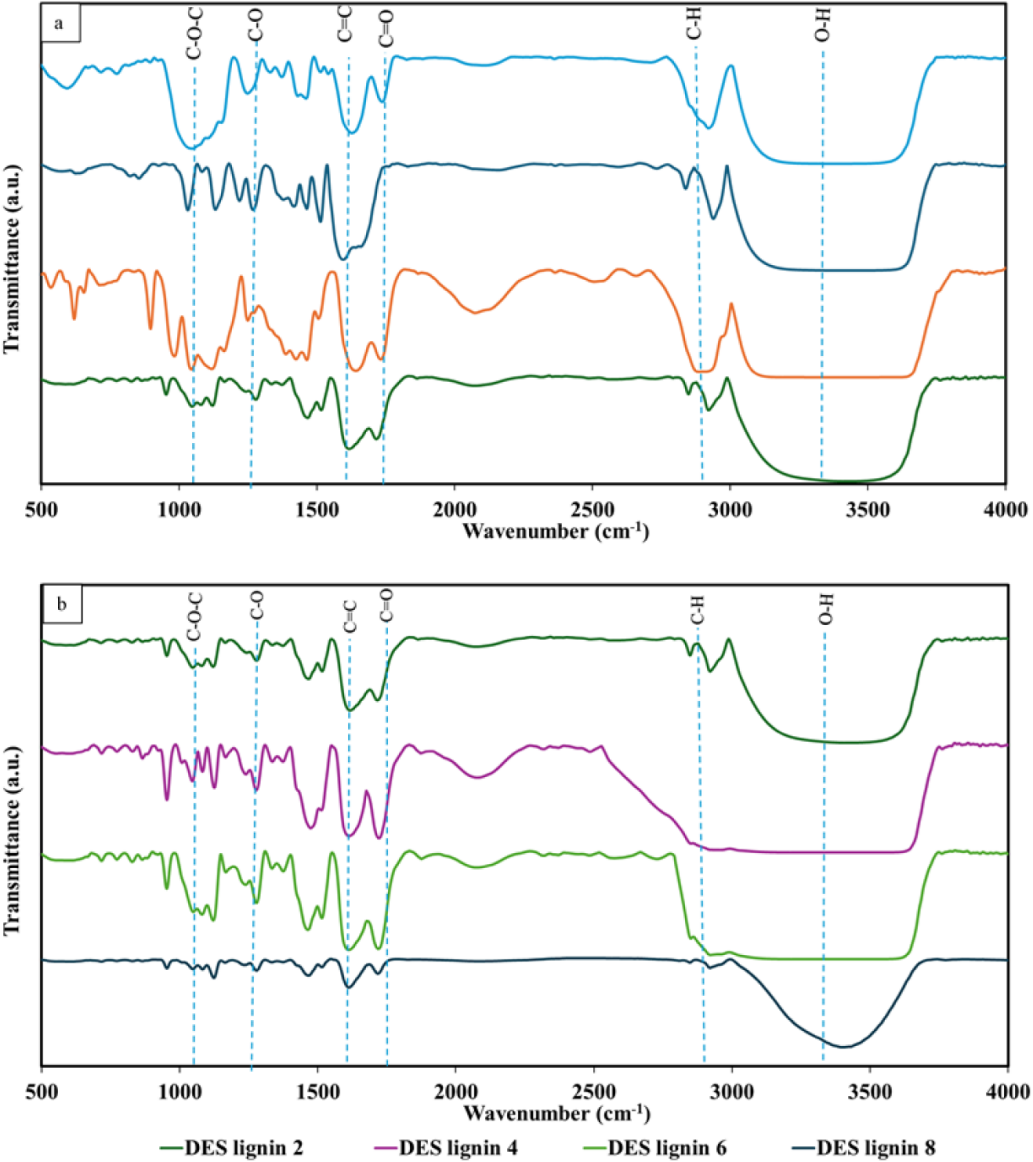
Fourier-transform infrared spectra for (a) DPW, commercial lignin, alkaline lignin, DES lignin 2, and (b) DES lignin extracted at different pretreatment times (2–4–6–8 h).

Figure 3b evaluates the effect of the pretreatment time on the infrared spectral distributions of DES lignin. The −OH bond stretches are broad, and the labels in Figure 3 are just indicative of their potential wavelengths. In fact, FTIR spectra indicate that the peak intensity of the O–H stretching band in the range of 3200–3500 cm^–1^ is reduced—particularly under 8-h-long pretreatment—suggesting a reduction in free hydroxyl groups due to the extended exposure of the sample to the DES.^31^ The C=O stretching peaks at 1700 cm^–1^ are more intense under 4- and 6-h-long pretreatment times, indicating increased oxidation and carbonyl-containing structure formation as the pretreatment time increases. The aromatic skeletal vibration peaks observed at 1600 cm^–1^ exhibit increased broadening as the pretreatment time increases, reflecting the progressive cleavage of aromatic linkages, particularly for the sample pretreated for 8 h.

These results demonstrate that DES lignin retains higher structural integrity at shorter pretreatment times. However, long pretreatment times of 6 and 8 h cause notable oxidative changes and aromatic linkage cleavage, possibly increasing the cleavage of internal bonds in lignin and disrupting its side-chain structural functional groups, as evidenced by FTIR spectra.^60^ This agrees as well with the lower lignin purity observed at pretreatment times greater than 4 h.

### 3.3 Morphological Characterization of Extracted Lignin

The SEM images are used to analyze the surface morphology of DPW, commercial lignin, alkaline lignin, and DES lignins extracted at different pretreatment times to obtain detailed insights into the structural characteristics of these samples, as shown in Figure 4. Figure 4a shows the prominent cylindrical fibers of the lignocellulosic biomass in DPW, where cellulose is enveloped by a monolayer of hemicellulose and embedded within a matrix of hemicellulose and lignin. The background of the fibers present in DPW comprises pectin, waxy materials, and other materials that act as a protective surface.^61^ Figure 4b illustrates that commercial lignin appears as agglomerates, with large particle-size variations and distribution. This irregularity is typical of industrial lignin and reflects the heterogeneity introduced during high-temperature pulping and precipitation processes. Figures 4c,d show the alkaline- and DES-extracted lignins, respectively. Both pretreatment processes eliminate the fibrous structure typically found in untreated biomass. Figure 4 also indicates that different extraction methods produce varying surface morphologies of lignin. The original structure of biomass shown in Figure 4a is broken down, and the lignin–hemicellulose matrix within the cell wall is degraded due to pretreatment.^62^ The DES lignin exhibits a smaller particle size than the alkaline-extracted lignin and more homogeneous lignin fragments, consistent with the DES lignin images presented in an earlier study.^63^ Moreover, alkaline lignin particles appear as irregular, fragmented clusters, as they are subjected to the strong alkali conditions during pretreatment that contribute to extensive breakdown of the lignin–carbohydrate complex, resulting in the formation of lower molecular weight fragments, as supported by molecular weight analysis. The more structured and less fragmented morphology of DES lignin indicates that the DES pretreatment method preserves the integrity of lignin to a greater extent. The reduced fragmentation of the DES lignin may also indicate better preservation of its macromolecular structure, increasing purity.

**Figure 4:**
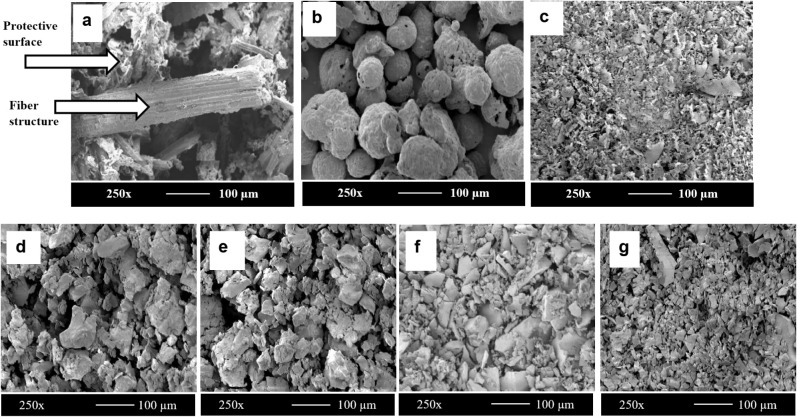
Scanning Electron Microscope images at 250× magnification and 100 μm scale for (a) date-palm waste (DPW), (b) commercial lignin, (c) alkaline lignin, and (d) e, f, g) DES lignin at 2, 4, 6, and 8 h of pretreatment, respectively.

The effect of a DES pretreatment time of 2 h on biomass is examined. The images presented in Figure 4d show coarse and irregular aggregates of lignin, with limited fragmentation. This corresponds to the early stages of delignification, where a notable portion of the lignocellulosic matrix remains intact. Increasing the pretreatment time to 4 and 6 h reduces the particle size, suggesting that lignin extraction is increased due to the improved dissolution of nonlignin components and more efficient lignin removal. Excessive fragmentation occurs under 8 h pretreatment, creating highly disrupted fine particles; this indicates the potential degradation of the lignin molecular structure due to excessive cleavage of interunit linkages within lignin, resulting from overexposure to the acidic environment. This excessive degradation observed in the SEM images after prolonged pretreatment times aligns with the purity and molecular weight results of lignin.

### 3.4 Determination of the Average Molecular Weight of Lignin

The average molecular weight of the commercial lignin was determined based on [η] to measure the molecular weights of the lignin samples. The molecular weight of the commercial lignin is 10.49 kDa, aligning precisely with a previously reported value.^64^ Figure 5 shows the average molecular weights (M_wt_) of the lignin samples estimated based on [η]. The molecular weight decreases gradually from commercial lignin to alkaline lignin to DES-extracted lignin. The commercial lignin exhibits the highest molecular weight of 10.49 kDa, while the alkaline lignin and DES lignin 2 display values of 8.83 and 7.85 kDa, respectively. As the pretreatment time increases to 4, 6, and 8 h, the molecular weights of the corresponding DES lignins decrease to 6.01, 4.93, and 4.90 kDa, respectively.

**Figure 5:**
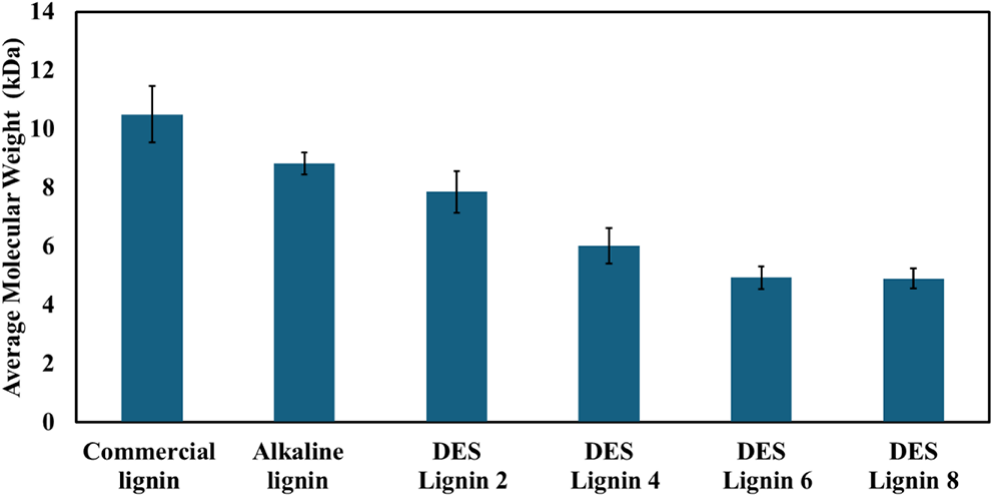
Average molecular weight for commercial lignin, alkaline lignin, and DES lignin extracted at different pretreatment times of 2, 4, 6, and 8 h. Error bars show the standard deviation of triplicate samples.

The higher molecular weight of the alkaline lignin compared to the DES lignin can be attributed to its low purity and the presence of cellulose and hemicellulose fractions, which have noticeably higher molecular weights than lignin.^65,66^ The lower molecular weight of 7.85 kDa observed for DES lignin 2 is consistent with the molecular weight of 7.34 kDa reported for lignin derived from marine-pine sawdust utilizing Lact:Tart:ChCl as the DES. A low molecular weight can be advantageous during subsequent processing into important bioproducts and for use in different applications.^67^ Moreover, a decrease in the lignin molecular weight with increasing pretreatment time confirms that the lignin undergoes depolymerization through ether bond cleavage as the pretreatment time increases, causing lignin fragmentation at high pretreatment times.^50,68^

### 3.5 Thermal Characterization of the Extracted Lignin Using TGA and DSC

The TGA of the extracted lignin samples, along with the DPW and commercial lignin samples, was performed to study their thermal stability and degradation temperatures. The presence of three degradation profiles in Figure 6 indicates the existence of three decomposition phases. The first stage occurs up to 250 °C, where moisture evaporation takes place. The second stage involves the notable degradation of the sample in the range of 250 °C–500 °C. This stage corresponds to the breakdown of interunit linkages, such as the C–O–C and C–C bonds, and the resulting production of phenolic compounds, gases, and other volatile chemicals.^69^ In this phase, commercial lignin preserves ∼65% of its initial weight at 500 °C, while the lignin extracted using DES retains ∼40%. Meanwhile, the lignin obtained via the alkaline pretreatment exhibits greater mass loss and retains only ∼25% of its initial weight. This suggests that most lignin components volatilize by 500 °C as the ether and ester linkages are cleaved.^70^ In the last stage, between 500 and 700 °C, aromatic rings undergo thermal breakdown, producing char or coke. While the alkaline-extracted lignin demonstrates a greatly lower residual char of <10%, the DES-extracted lignin displays a residual char level of 21%. For the commercial lignin, DES-extracted lignin, and alkaline-extracted lignin, the maximum derivative thermogravimetry peak (DTG_max_) values are observed at roughly 360 °C, 273 °C, and 236 °C, respectively (Figure 6b).

**Figure 6:**
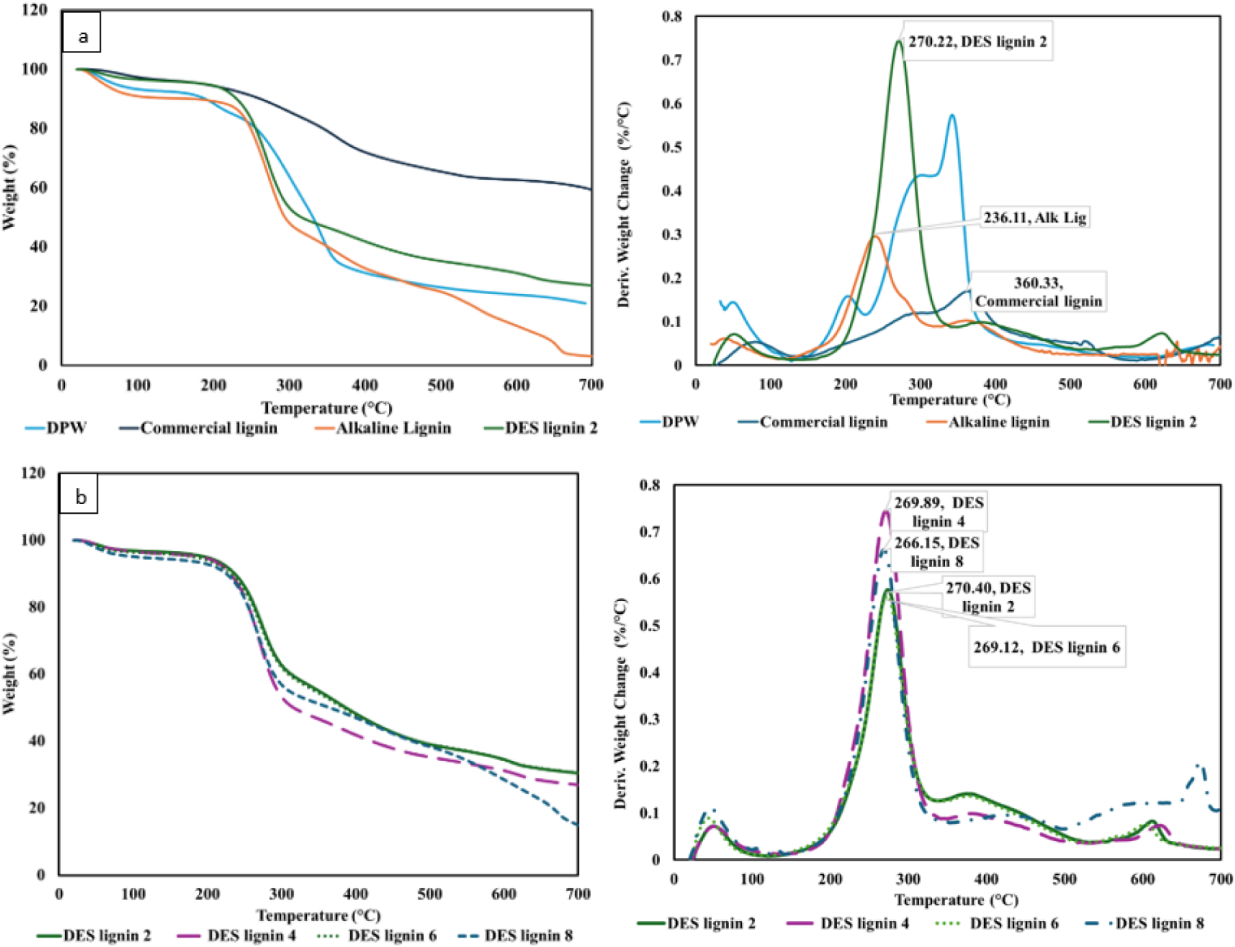
TGA and DTG_max_ curves for (a) DPW, commercial lignin, alkaline lignin, DES lignin 2 and (b) DES lignin extracted at different pretreatment times of 2, 4, 6, and 8 h.

When comparing lignin extracted with different extraction methods, DES lignin consistently shows higher thermal stability than alkaline lignin, as presented in both the TGA and DTG curves in Figure 6. The alkaline lignin starts to degrade at lower temperatures and shows a DTG_max_ of only 236 °C, indicating the presence of a more degraded and fragmented structure. This reduced stability is likely due to the severe chemical conditions of the alkaline process, which can break internal ether bonds, reduce cross-linking, and lead to a more condensed but less thermally stable lignin.^71^ In contrast, DES lignin has a higher thermal stability than DES lignin, indicating a more intact aromatic structure with less severe cleavage of internal bonds. Additionally, this can be ascribed to its higher purity,^57^ as the impurities in lignin samples can substantially affect the thermal characteristics of extracted lignin.^72^

The TGA curves of the DES lignin samples extracted at different pretreatment times show only slight differences in their thermal behavior, although the DES lignin pretreated for 8 h displays a significantly increased weight loss. This can be attributed to its lower molecular weight, as lower-M_wt_ lignin may contain more volatile or lower-stability components, leading to greater mass loss upon heating. The DTG curves exhibit noticeable differences in DTG_max_ values, as shown in Figure 6b, with the DES lignin pretreated for 2 h displaying the highest value at 270.4 °C and that pretreated for 8 h demonstrating the lowest value at 266 °C. This downward trend in DTG_max_ with increasing pretreatment time indicates a gradual reduction in thermal stability, correlating with the formation of smaller, more volatile lignin fragments due to prolonged chemical exposure.

Figure 6b illustrates the main degradation stages of DPW, showing that thermal degradation occurs in multiple stages due to the varying weight percentages of lignin, hemicellulose, and cellulose. These biopolymers transition into their amorphous and crystalline phases at different temperatures. Compared to alkaline lignin, the elevated DTG_max_ (temperature at the maximum degradation rate) observed for the DES lignin, as presented in Table 1 confirms its higher thermal stability. The DTG curves of the DES lignins extracted at different pretreatment times show that DTG_max_ decreases as the pretreatment time increases, indicating a decrease in the thermal stability of these lignin samples. Higher degradation temperatures observed for the lignin pretreated for 2, 4, and 6 h indicate that they have more stable and less fragmented structures. Meanwhile, the lower DTG_max_ value noticed for the lignin pretreated for 8 h implies that it has lower stability, which may be caused by the presence of more fragmented, modified structures with a lower molecular weight resulting from depolymerization at a higher pretreatment time.^57^

**Table 1:** TGA and DTG Analyses for DPW, Commercial Lignin, Alkaline Lignin, and DES Lignin Extracted at Different Pretreatment Times of 2, 4, 6, and 8 h

Sample	Weight Loss at 250 °C (%)	Weight Loss at 700 °C (%)	T at 50% weight loss (°C)	DTG_max_ (°C)
DPW	81.35	20.97	331.34	
Commercial lignin	91.07	59.30	Not in range	360.33
Alkaline lignin	79.56	3.04	294.48	236.10
DES lignin at 2 h	86.251	30.74	386.193	270.40
DES lignin at 4 h	85.132	30.55	316.01	269.88
DES lignin at 6 h	83.13	26.94	382.9	269.11
DES lignin at 8 h	82.68	14.89	364.7	266.14

The observed thermal stability trends in the TGA and DTG data are consistent with the molecular weight measurements. The commercial lignin, with the highest molecular weight (10.49 kDa), exhibits the highest DTG_max_ (∼360 °C) and greatest residual mass, indicating a more thermally stable structure. In contrast, the DES-extracted lignins show a gradual decrease in both molecular weight (from 7.85 to 4.90 kDa with increasing pretreatment time), which is in accordance with mass losses observed by TGA and also the DTG_max_ (from 270.4 to 266.14 °C), suggesting that depolymerization during prolonged pretreatment produces smaller, more fragmented lignin molecules that degrade at lower temperatures. Similarly, the alkaline lignin, despite a relatively higher molecular weight (8.83 kDa) than some DES fractions, displays lower thermal stability (DTG_max_ ∼ 236 °C), likely due to the presence of impurities and partial fragmentation caused by severe alkaline conditions. These correlations indicate that higher molecular weight lignin fractions generally correspond to increased thermal stability, while lower molecular weight or more fragmented lignins are more prone to decomposition, providing complementary support for the TGA observations.

The DTG_max_ values reflect the structural differences between the DES- and alkaline-extracted lignins. DES lignins exhibit higher DTG_max_ values (∼270 °C) compared to alkaline lignin (∼236 °C), indicating a more intact aromatic structure with less fragmentation. In contrast, the lower DTG_max_ of alkaline lignin is consistent with greater cleavage of internal ether bonds and the presence of impurities, resulting in a more degraded and thermally labile structure.

DSC was performed to analyze thermal transitions occurring upon heating in commercial lignin and alkaline- and DES-extracted lignins. DSC analysis is illustrated in Figure 7, and the results are presented in Table 2. The thermal parameters extracted from the DSC spectra are used to determine the glass transition temperatures T_g_ and melting temperatures T_m_. Figure 7 shows that the thermal profiles are different for various samples, suggesting differences in their thermal behaviors and purity. All DSC spectra exhibit similar trends for temperatures below 50 °C due to moisture loss.^32^ As the temperature increases, the commercial lignin displays a substantial endothermic transition at approximately 131 °C. Furthermore, the DES lignin 2 exhibits a gradual endothermic peak at 162 °C, while the alkaline lignin displays a broad endothermic transition peak between 80 and 130 °C. This peak indicates that the alkaline lignin structure is more disturbed and fragmented. In addition, an exothermic process occurs in the alkaline lignin when the temperature exceeds 200 °C, likely due to its lower purity.

**Figure 7:**
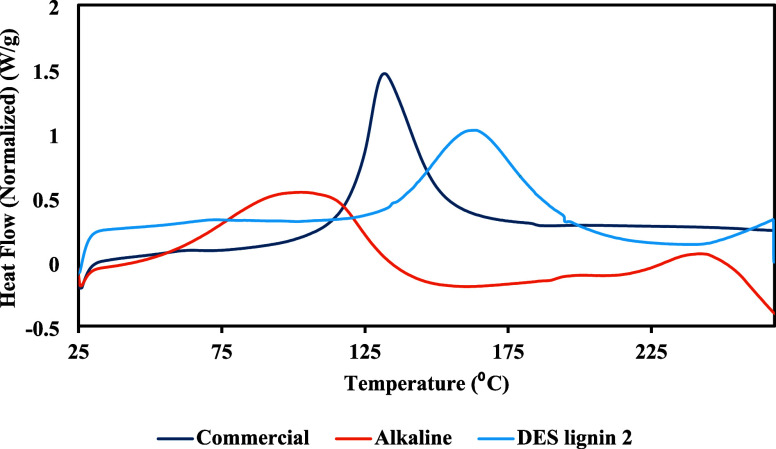
Thermograms from the DSC of commercial lignin, alkaline lignin, and DES-extracted lignin at 2 h. The endothermic direction is upward.

**Table 2:** DSC Results for Commercial Lignin, Alkaline Lignin, and DES-Extracted Lignin

Sample	T_g_ (°C)	T_m_ (°C)	Enthalpy ΔH_m_ (J/g)
Commercial lignin	57.96	131.71	155.2
DES lignin	65.69	163.61	141.8
Alkaline lignin	59.29	101.82	66.39

The T_g_ values of the commercial, alkaline, and DES lignins are reported in Table 2. A higher glass transition temperature of the DES lignin indicates its more rigid structure, possibly due to the preservation of its aromatic structure during DES pretreatment.^73^ Additionally, the enthalpy of melting determined from DSC, representing the amount of heat a material absorbs during melting, implies that the commercial lignin has a higher enthalpy of melting (155.2 J/g) than the DES-extracted lignin (141.8 J/g) and that the alkaline lignin exhibits a much lower enthalpy of melting (66.39 J/g). The enthalpy of melting observed for the DES lignin in this study is significantly higher than the enthalpy of 130.2 J/g reported for lignin extracted from kenaf-fiber biomass using ChCl:LA as the DES.^32^ The higher enthalpies of melting for the commercial lignin and DES lignin signify that additional energy is necessary to break bonds in these lignin structures.^74^

The thermal analysis results demonstrate that the DES-extracted lignin exhibits greater thermal stability than the alkaline lignin. The elevated degradation temperatures and residual char concentration exhibited by the DES lignin—as evidenced from the TGA results—indicate that it has a more intact structure with enhanced resistance to thermal degradation. The DSC analysis thus reveals the thermal transition behavior, showing that DES lignin exhibits an elevated glass transition temperature and enthalpy of melting, implying a more rigid molecular structure that requires greater energy for thermal reactions. By contrast, the alkaline lignin exhibits reduced enthalpy of melting due to its fragmented structure caused by extensive pretreatment.

### 3.6 Crystallographic Structure Analysis Using XRD

The XRD spectra of raw DPW, commercial lignin, alkaline lignin, and DES lignin, shown in Figure 8, reveal their crystalline structures. The DPW spectrum clearly shows distinct peaks at around 16° and 22°. According to the literature, these peaks match the (101) and (002) crystallographic planes, respectively, which define the natural structure of cellulose.^75^ The XRD spectrum of the commercial lignin displays a broad signal at 2θ = 22°, typically indicative of the predominantly amorphous nature of lignin.^51^ The XRD patterns of the alkaline- and DES-extracted lignins display reduced peak intensities, suggesting that the highly crystalline structure of cellulose is removed. Notably, the alkaline lignin exhibits some distinct peaks at around 19°, 23°, 31°, and 35°, attributed to residual crystalline cellulose^51^ and hemicellulose,^62,76^ validating the purity results reported earlier. In addition, Figure 8b illustrates the spectra of lignins obtained by using DES pretreatment for different times. This figure shows a consistent overall spectral pattern with small differences, indicating that the DES-treated lignin mostly retains its amorphous properties; however, at higher pretreatment times, the spectra show lower peak intensities, possibly suggesting the extensive degradation of lignin particles at these pretreatment times.

**Figure 8:**
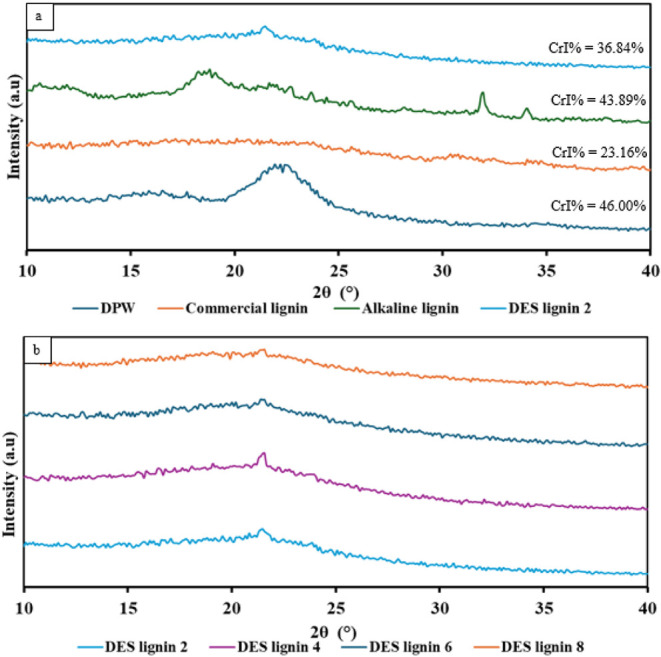
X-ray diffraction spectra for (a) DPW, commercial lignin, alkaline lignin, DES lignin 2 and (b) DES lignin extracted at different pretreatment times (2–4–6–8 h).

Among all samples, the raw DPW demonstrates the highest CrI% value of 46%, which indicates the presence of native cellulose and hemicellulose.^77^ This result aligns with the reported CrI% range of 42.44%–57.96% for raw lignocellulosic biomass. A low CrI% value observed for commercial lignin indicates that it mainly has an amorphous structure, whereas a high CrI% of 43.89% for alkaline lignin signifies the incomplete elimination of crystalline cellulose and hemicellulose during pretreatment. This is consistent with the purity results, as the alkaline lignin shows just a 5% increase in lignin content compared to that of the raw DPW. The DES lignin displays reduced crystallinity of 36.84% relative to that of the alkaline lignin, suggesting that DES pretreatment results in better lignin extraction and purity.

### 3.7 Phenolic Hydroxyl Content of Extracted Lignin

The phenolic hydroxyl group is the reactive functional group in the lignin structure, and its determination is essential to provide additional information about lignin properties as it assesses the reactivity of lignin.^78^ The results highlighted that lignin’s PhOH content is greatly dependent on the biomass source and extraction conditions.^42^ The results revealed that commercial lignin had the highest PhOH content at 2.46 mmol/g, followed by the DES-extracted lignin at 4 h at 2.09 mmol/g, and the alkaline-extracted lignin at 1.70 mmol/g. These results are in agreement with the phenolic hydroxyl content reported in the literature, as kraft spruce lignin was reported to have 2.62 mmol/g.^79^ Additionally, lignin extracted from *Eucalyptus grandis* using Lact:ChCl DES was reported to have PhOH ranging from 0.69 to 2.10 mmol/g.^80^ The phenolic hydroxyl group content of lignin extracted from bamboo using DES (oxalic acid/choline chloride) (1:1) at 160 °C for 120 min was 1.871 mmol/g.^78^ The elevated PhOH value of the DES lignin compared to alkaline lignin could be explained by the DES system’s more substantial hydrogen-bonding and acidity, which may favor the cleavage of β-O-4 ether bonds over ester formation, leading to increased phenolic hydroxyl groups in isolated lignin.^81^ In contrast, the lower PhOH content of the alkaline-extracted lignin may reflect milder cleavage of ether linkages and a lower reactivity due to condensation reactions.^82^ These findings indicate that both commercial and DES lignins possess higher phenolic functionality, making them more suitable for reactive applications.

### 3.8 Free-Radical Content from ESR Spectroscopy

ESR spectroscopy of the raw DPW, commercial lignin, and lignin extracted from DPW using alkaline and DES pretreatments can provide valuable insights into the presence, concentration, and type of free radicals in different samples and can be used to evaluate their radical stability and extent of degradation. The free radical concentrations and g-values of the samples are reported in Table 3. The results indicate that the raw, untreated DPW has the lowest free radical concentration, which aligns with previous literature.^83^ Free radical concentration is higher in lignin compared to cellulose and hemicellulose;^84^ however, lignin makes up only 17% of the raw untreated DPW. A higher concentration of free radicals in commercial lignin, probably resulting from industrial manufacturing processes, can produce additional radical species. Furthermore, the DES lignin exhibits a greater free-radical concentration than the alkaline lignin; the pretreatment temperature is reported to play a significant role, as it assists in bond cleavage within the structure. Since DES and alkaline lignin were subjected to 120 and 80 °C, respectively, there is higher homolytic bond cleavage within weak covalent bonds that led to the formation of additional radicals present in DES lignin.^83^ *g*-value results showed that both extracted lignins had *g*-values below 2.0045. The variation in the *g*-factors is possibly a consequence of the differing acidity in the extraction mixtures, which may influence the protonation states of the semiquinone radicals or lead to the predominance of different radical species.^85^

**Table 3:** ESR Analysis for Commercial Lignin, Alkaline Lignin, and DES-Extracted Lignin

Sample	Free-Radical Concentration (spins)	*g*-Value
DPW	1.568 × 10^15^	2.00432
Commercial lignin	2.22 × 10^17^	2.00482
Alkaline lignin	3.141 × 10^15^	2.00414
DES lignin	4.520 × 10^15^	2.00440

### 3.9 Industrial Implications of the Green Extraction of Lignin from Date Palm Waste

This study investigated the extraction of lignin from DPW using alkaline and DES pretreatment methods. Although alkaline pretreatment achieved a higher lignin yield, the study demonstrated that it resulted in a lower purity of 27.42% because of the coextraction of impurities, such as hemicellulose and cellulose. Meanwhile, DES pretreatment, particularly under a 4-h-long pretreatment time, resulted in a balance of good yield and significantly higher purity of 63.44%. From an industrial perspective, these findings present valuable opportunities for advancing the valorization of lignocellulosic biomass. Although alkaline extraction is the most commonly used approach for lignin, the existence of a large number of residual carbohydrates necessitates additional purification processes.^86^ Given the versatility of lignin as an important raw material for many applications in adhesives,^87^ rechargeable batteries,^88^ biocomposites,^89^ thermal insulators,^90^ and others^91,92,93^ improving the extraction processes is necessary. The use of DES pretreatment for lignin extraction not only increases purity but also provides a more sustainable and eco-friendly approach compared to traditional alkaline methods.^94^ Moreover, the scalability of DES pretreatment, together with its low toxicity, versatility, improved solubility, and reduced environmental impact,^95^ aligns with the global shift toward green industrial practices.

## 4 Study Limitations and Industrial Scalability

While this study provided a comparative analysis of alkaline and DES pretreatments for lignin extraction from date-palm waste, along with comprehensive characterization to evaluate the structural and thermal properties of the extracted lignin, certain limitations should be acknowledged. Notably, the study lacked advanced structural characterization techniques such as nuclear magnetic resonance (NMR) and gel permeation chromatography (GPC), which would provide deeper insights into lignin’s molecular structure and weight distribution. Due to equipment constraints, structural interpretations depended on FTIR, TGA, DSC, XRD, and SEM, which, although beneficial, are more indicative than definitive regarding lignin structure. Furthermore, this study was primarily focused on the extraction and detailed characterization of lignin from date-palm waste using alkaline and DES pretreatments. While a complete mass balance of all biomass fractions was not determined, future work should aim to include full component recovery analysis to provide a more comprehensive assessment of the process efficiency and integration potential.

Deep eutectic solvent pretreatment presents an environmentally beneficial method to enhance the economic value of lignin. The utilization of deep eutectic solvents (DESs), composed of inexpensive, biodegradable, and low-toxicity substances^95^ such as choline chloride and formic acid, aligns with green chemistry principles, enhancing the attractiveness of this extraction approach for industries. This is particularly relevant in places such as the Middle East and North Africa, where DPW is produced in large amounts as agricultural waste.

The DES pretreatment method demonstrated in this study enabled the recovery of high-purity lignin with favorable thermal and structural characteristics, making it a strong candidate for downstream industrial applications, such as thermal insulation foams, biobased polymers, and additives. However, despite its laboratory-scale success, several key challenges must be addressed to enable industrial scalability, such as affordability and solvent recovery. The method employs neither harsh chemicals nor high-pressure systems; yet, the expense of DES, particularly in larger quantities, remains a concern. The technology could be significantly more cost-effective with improved solvent recycling methods and the development of bio-DES with components derived from biomass. Bio-DES will not only reduce costs but also align more effectively with the sustainability objectives of integrated biorefineries. Additionally, the current process uses a lot of energy because it involves heating, filtering, washing, and freeze-drying. Adding DES extraction to a biorefinery that makes more than one product and shares waste heat and infrastructure could help the whole process use less energy and reduce costs . Despite these challenges, the superior performance of DES-extracted lignin in terms of purity, thermal stability, and structural integrity indicates significant economic potential. Continued investigation into solvent recycling methodologies and techno-economic assessments may enable the large-scale application of DES-based lignin extraction from DPW in future lignin extraction systems.

## 5 Conclusions

This study enhances our understanding of employing DES pretreatment for lignin extraction from date-palm wood and establishes it as a more efficient and green lignin extraction process, especially when compared to alkaline pretreatment. Several conclusions are elucidated in this study.

1. DES pretreatment yields lignin from DPW with up to 63.44% purity, compared to 27.42% for alkaline extraction. DES processing allows selective lignin extraction with less contamination, as well as more structural integrity and thermal stability.
2. Higher pretreatment times lead to lignin degradation, decreasing the yield and purity. The optimum DES pretreatment time was found to be 4 h with a yield of 22.85% and a purity of 58.89%.
3. DES-extracted lignin has a smaller molecular weight (7.85 kDa) and a higher degradation temperature (270 °C) than alkaline lignin (236 °C), indicating improved thermal stability. These properties suggest better polymer synthesis and thermal insulation compatibility.

This study has remarkable implications, as it offers a feasible route for converting abundant DPW into high-value bioproducts, thereby supporting a circular bioeconomy and encouraging environmentally acceptable methods for lignin-derived material production. Future work should focus on DES recycling, energy efficiency, and process scale-up to bridge the gap between the lab and industry.

## Data Availability

The data supporting this study are available within the manuscript.

## Abbreviations

AIL: Acid-Insoluble Lignin
ASL: Acid-Soluble Lignin
DES: Deep Eutectic Solvent
DPW: Date-Palm Waste
DSC: Differential Scanning Calorimetry
ESR: Electron Spin Resonance
FA: Formic Acid
FTIR: Fourier Transform Infrared Spectroscopy
LA: Lactic Acid
NREL: National Renewable Energy Laboratory
SEM: Scanning Electron Microscopy
TAPPI: Technical Association of the Pulp and Paper Industry
TGA: Thermogravimetric Analysis
UV–vis: UV–Visible
XRD: X-ray Diffraction

## Glossary

Deep Eutectic Solvent (DES): A type of green solvent formed from a mixture of a hydrogen bond donor (HBD) and a hydrogen bond acceptor (HBA) which forms a eutectic with a lower melting point.
Thermogravimetric Analysis (TGA): A technique to assess material stability by measuring weight loss with increasing temperature.
Differential Scanning Calorimetry (DSC): A method used to study the thermal transitions that occur in materials as they are heated (e.g., melting, glass transition).
Scanning Electron Microscopy (SEM): Imaging technique for observing morphology and surface structure.
Fourier-Transform Infrared Spectroscopy (FTIR): Analytical method used to identify functional groups in samples.
Acid-Insoluble Lignin (AIL): Lignin fraction that precipitates in acidic conditions.
Acid-Soluble Lignin (ASL): Lignin fraction that remains dissolved in acidic filtrate, quantified via UV–vis spectroscopy.
Valorization: The process of converting waste materials into more valuable products.
